# Comparison of cycle threshold values of the Cobas HPV test and viral loads of the BMRT HPV test in cervical cancer screening

**DOI:** 10.3389/fpubh.2022.1010066

**Published:** 2022-11-10

**Authors:** Qing Yang, Hui Du, Xinfeng Qu, Wenkui Dai, Liming Gui, Changzhong Li, Chun Wang, Chunlei Guo, Yi Zhang, Lihui Wei, J. L. Belinson, Ruifang Wu

**Affiliations:** ^1^Department of Obstetrics and Gynecology, Peking University Shenzhen Hospital, Shenzhen, China; ^2^Institute of Obstetrics and Gynecology, Peking University–Hong Kong University of Science and Technology (PKU-HKUST) Medical Center, Shenzhen, China; ^3^Shenzhen Key Laboratory on Technology for Early Diagnosis of Major Gynecologic Diseases, Peking University Shenzhen Hospital, Shenzhen, China; ^4^Department of Obstetrics and Gynecology, Peking University People's Hospital, Beijing, China; ^5^Preventive Oncology International, Inc., The Women's Health Institute, Cleveland Clinic, Cleveland, OH, United States

**Keywords:** human papillomavirus, cervical cancer, Cobas4800 assay, BMRT assay, viral loads

## Abstract

**Objective:**

To validate the HPV viral loads that are reflected by the cycle threshold values of Cobas4800 as the viral load indicators by verifying the consistency of the viral loads per unit (10,000 cells) from the BMRT assay.

**Methods:**

The analysis is based on data from the Chinese Multi-Center Screening Trial (CHIMUST). The cases included in the analysis are all positive for physician-collected hrHPV on SeqHPV and/or Cobas4800 or negative for hrHPV but abnormal in cytology (≥LSIL), and some cases selected by nested case-control randomization from those negative for physician-collected hrHPV and cytology. With HPV testing results and relevant Ct values from Cobas4800 available, we tested the entire sample set with the BMRT HPV testing assay and analyzed their agreement with Cobas4800, followed by a comparison of the CtV from Cobas4800 and viral loads (lg) from BMRT by lesion grade.

**Results:**

We included 4,485 women (mean age: 45.4 years) in the study, and 4,290 had complete data. The consistency of genotypes from Cobas4800 and BMRT for hrHPV, HPV-16, HPV-18, and 12-HPV pools was 94.9% (4070/4290, Kappa = 0.827), 99.1% (4251/4290, Kappa = 0.842), 99.6% (4,273/4,290, Kappa = 0.777), and 95.3% (4,089/4,290, Kappa = 0.821), respectively. Further analysis shows that any inconsistency between the two assays is likely among samples with comparatively lower viral loads. When analyzing per lesions of CIN2+ and CIN3+, the CtV from Cobas4800 and VL (lg) from BMRT are highly correlated inversely and follow the linear regression for HPV16 and 12-HPV pool (Pearson's or Spearman's correlation coefficient (r): In CIN3+, r _HPV16_ = −0.641, *P* < 0.001; r _12−HPVpool_ = −0.343, *P* = 0.109; In CIN2+, r _HPV16_ = −0.754, *P* < 0.001; r _12−HPVpool_ = −0.429, *P* < 0.001).

**Conclusion:**

The CtV from Cobas4800 and the viral loads (lg) of per unit cells from the BMRT are well correlated for lesion grading when tested on physician-collected samples. Cobas-CtV is worthy of further study for clinical application.

## Introduction

Cervical cancer is the fourth most common cancer in women. In 2020, an estimated 604,000 new cases of cervical cancer were found, and there were 342,000 deaths worldwide (1). The incidence and mortality of cervical cancer in China are much higher than in developed countries (2). However, cervical cancer is the only cancer with a clear etiology, and it is preventable with HPV vaccination, early detection, and effective pre-cancer management. There is a consensus that persistent infection with high-risk human papillomavirus (hrHPV) is the fundamental cause of the development of cervical cancer (3). In 2021, global leaders called for cervical cancer elimination (4). In China, the world's most populous country, the newly introduced free vaccination program for school-aged girls with the domestic bivalent HPV vaccine and the national free cervical cancer screening plan play a significant role in the prevention of cervical cancer.

Currently, the coverage of cervical cancer screening in China is low and needs to be increased with effective technologies and efficient implementation. Compared with cytology, which is highly resource-dependent, has relatively low sensitivity, and is very skill-dependent, HPV testing for primary screening is an objective technology, higher in sensitivity and negative predictive value (NPV), and is very reproducible (5). Consequently, HPV testing is becoming the dominant method for primary cervical cancer screening (5). Self-collected samples have been validated for a long time to be comparable to physician-collected specimens in sensitivity and specificity when tested on PCR-based HPV assays. They are now recommended for population-based screening programs to rapidly widen coverage (6).

HPV testing has a significant disadvantage: specificity for detecting CIN2/3. Because most HPV infections are non-neoplastic, this creates challenges for risk stratification and the need for secondary screening (7). Cytology and colposcopy are commonly used in high-resource screening programs for secondary screening and diagnostic triage. These technologies depend on the experience and knowledge of cytologists and the doctors performing colposcopy, which are scarce resources for cervical cancer control in low- and middle-income countries (LMIC). Several studies have shown the association of carcinogenic risk with HPV genotypes and viral load. Some studies have demonstrated that HPV genotype and viral load can be used for risk stratification and managing patients testing HPV-positive (8–10). The Molc-Model, which integrates molecular technologies alone, has been tested and applied to secondary screening and triage (8–10). Application of the Molc-Model would enable cervical cancer screening to be set in the primary medical facilities near the community women who need screening. It would significantly reduce the dependence on experienced colposcopy doctors. The widespread application of such a model for LMICs could substantially expand cervical cancer screening and triage worldwide.

Currently, there are about 250 HPV tests worldwide, most of them PCR-based assays in China. PCR-based assays can report the cycle threshold value (CtV). However, only a small number of assays have been clinically validated (11). The Cobas4800, one of the most commonly used HPV testing assays, was the first HPV test FDA-approved for primary screening in women (≥25 years of age). Cobas4800 is a semi-quantitative HPV test assay for HPV-16, HPV-18 individually, and 12 other hrHPV types pooled. It can report the CtV, reflecting the number of amplification cycles needed to complete a particular sample's test. It inversely reflects the viral load of the specimen being tested and is dependent on the cell quantity in the specimen (12). Previous work from other authors has demonstrated that CtV from Cobas4800 is valuable for triage, especially when correlated with the HPV genotypes (13). However, direct evidence is required to demonstrate that CtV from the Cobas4800 can indicate per-specimen viral load.

The Bioperfectus Multiplex Real-Time (BMRT) HPV assay is the only PCR-based HPV testing assay that reports the genotype-specific viral load of 21 HPV genotypes per unit (defined as 10,000 cells) (14). We have validated in CHIMUST that the sensitivity and specificity of the BMRT assay for detecting CIN2+ lesions are statistically similar to Cobas4800 when limited to just the 14 hrHPV types in the Cobas4800 assay (10). With this in mind, we analyzed the concordance between CtV from Cobas4800 and the direct viral loads of BMRT.

## Materials and methods

### Study population

This study used physician-collected cervical samples from the Chinese Multi-center Screening Trial (CHIMUST). CHIMUST was a population-based cross-sectional study (Trial Registration Number: ChiCTR-EOC-16008456) conducted collaboratively from August 2016 to January 2018 by six hospitals in six provinces in northern, middle, eastern, and southern China (15). A total of 10,855 eligible women were enrolled in this trial. Each contributed a physician-collected sample and a self-collected sample. The CHIMUST protocol was approved by the Ethics Committee of Peking University Shenzhen Hospital (IRB: PUSH2016001) and the Cleveland Clinic Institutional Review Board (IRB: 15–1549).

The physician-collected samples were tested by the Cobas4800 (Roche, Pleasanton, CA, USA) and SeqHPV (BGI, Shenzhen, China) assays. Colposcopy-directed and random biopsies by quadrant were performed on all cases positive on either of the two primary testing assays. Using a nested case-control method, we performed BMRT HPV testing on the part of the stored physician-collected specimens from CHIMUST and compared the results with the Cobas4800. We evaluated the sensitivity and specificity for CIN2+, the concordance of CtV from Cobas4800 (CtV-Cobas), and the genotype-specific viral load reported by BMRT (VL-BMRT). We also analyzed the trends of the CtV-Cobas and the VL-BMRT by the pathologic grade of the confirmed cervical lesions. This CHIMUST sub-study was approved by the Ethics Committee of Peking University Shenzhen Hospital (IRB: PUSH2019044).

Non-pregnant women aged 30 to 59 with an intact uterus, no history of pelvic radiation or damaging cervical treatment, and no cervical cancer screening for 3 years were enrolled in CHIMUST. All participants provided informed consent to participate in the trial and for the future use of their samples and related personal de-identified information.

At the first participation visit, each participant obtained a vaginal sample by herself (self-collected sample). Then, the participant was escorted to another room for physician sampling following the standardized sampling procedures (rotating the brush three times after removing cervical secretions) to obtain exfoliated cervical cells from the transformation zone and the squamo-columnar junction of the cervix. The physician-collected cervical sample was placed into a vial containing 20 ml of PreservCyt^®^ solution (Hologic, Marlborough, Mass., USA) for HPV testing.

Both the self- and physician-collected samples were tested on the Cobas4800 and SeqHPV, while the physician-collected samples were also processed for cytology using ThinPrep by senior cytopathologists from PUSH (Hologic, USA). Participants who were positive for hrHPV on one or both of the testing assays (self- and/or physician-collected samples) were referred for colposcopy and biopsy plus endo-cervical curettage (ECC) (16).

**Figure 1:**
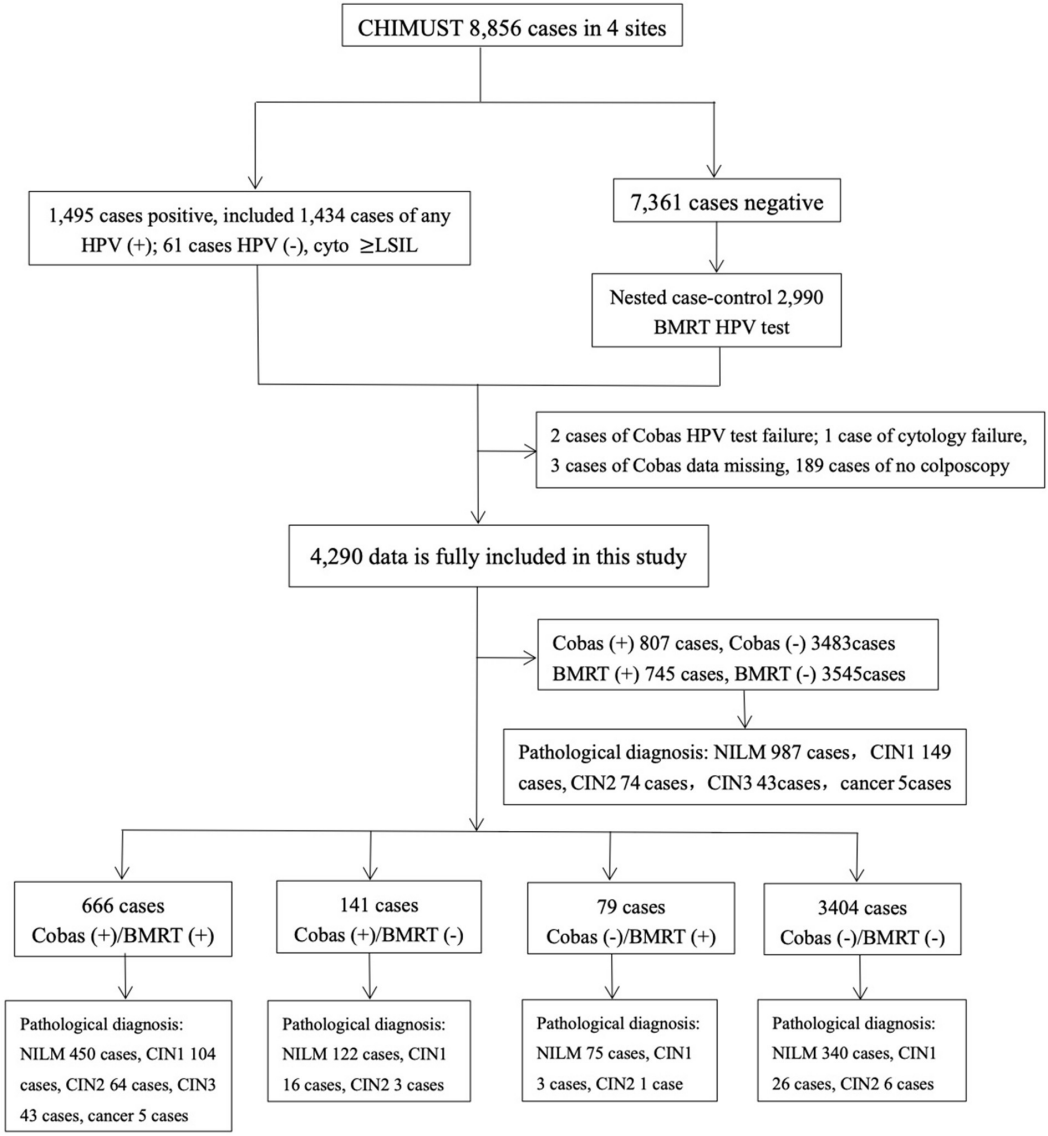
Study population.

This study was conducted based on 8,856 physician-collected samples from the screening sites in Beijing, Hebei, Hubei, Jiangxi, and Shanghai. Among those samples, 1,434 were hrHPV positive on SeqHPV and/or Cobas4800, and 61 samples were HPV negative but with cytology ≥LSIL. Therefore, a total of 1,495 samples were considered to be “positive samples.” We included the cases abnormal by cytology only for fair validation of BMRT's effectiveness in detecting CIN2+ cases. The nested case-control sample included all the positive samples and doubled that number for the matched negative samples by women's ages, sampling site, and sampling time. A total of 2,990 samples negative for HPV and cytology (the negative samples) were randomly picked, making a total of 4,485 samples to be the “sample set” for BMRT testing (Figure 1).

## Study methods

### Cobas4800

Cobas4800 is an HPV test based on real-time fluorescent PCR amplification technology. The specially provided equipment can automatically run the testing procedures, including sample preparation, DNA extraction, PCR amplification, and DNA detection for 14 hrHPV genotypes. It refers human β-globin gene in each run to monitor cell adequacy and to provide an internal control for positive and negative results. With four configured channels, Cobas4800 reports HPV-16, HPV-18, 12 types of hrHPV in pool (31, 33, 35, 39, 45, 51, 52, 56, 58, 59, 66, and 68), and β-globin. At the same time, it can report the CtV of each channel, which reflects the number of cycles of DNA amplification required in the PCR process to detect HPV DNA. The CtV is inversely correlated with the logarithm of the target DNA in the testing sample (17, 18). Therefore, a higher CtV reflects a lower viral load in the sample.

### BMRT HPV test

The BMRT HPV test is also based on real-time fluorescent PCR amplification technology. It detects 21 HPV genotypes, including 14 high-risk HPV genotypes (HPV-16, −18, −31, −33, −35, −39, −45, −51, −52, −53, −56, −58, −59, and −66), 4 mid-risk HPV genotypes (HPV-26, −68, −73, and −82) and 3 low-risk HPV genotypes (HPV-6, −11, −81). It performs eight reactions in eight tubes simultaneously per sample, in which HPV-16/-18/-31, HPV-59/-66/-53, HPV-33/-58/-45, HPV-56/-52/-35, HPV-68/-51/-39, HPV-73/-26/-82, and HPV-6/-11/-81 are tested in groups in tubes A, B, C, D, E, F, and G, respectively, leaving tube H to detect human topoisomerase III (human TOP3), a single-copy gene within a single human cell. Analytic software is applied to calculate the number of viral copies per 10,000 cells in the HPV-positive samples (the viral loads) *via* analyzing the linear relationship between the single-copy gene and the number of cells. The BMRT HPV test reports the viral loads for each of the 21 HPV subtypes. In this study, we refer to the viral loads of 14 hrHPV genotypes from BMRT to compare them with the CtVs of the same hrHPV genotypes from Cobas4800. The viral copy in 10,000 cells is reported together with the testing results of BMRT by genotype.

### Colpo/biopsy and histological diagnostics

All patients who returned for diagnosis had colposcopy and biopsies. We followed a POI protocol: multiple biopsies were performed directly at the lesion site (s) plus randomly at the squamocolumnar junction in other quadrants without visible lesions and endocervical curettage (ECC) for all patients (16). Peking University Shenzhen Hospital's senior pathologists in the Gynecological Pathology Lab processed and examined the biopsied specimens. The physicians who performed colposcopy were all blind to the cytology results. Pathological diagnoses were reported quadrantally as negative for intraepithelial lesion/malignancy (NILM), cervical intraepithelial neoplasia (CIN) grade 1 (CIN1), CIN2, CIN3, microinvasive cancer, and invasive cancer. The highest pathological grade among all quadrant-based biopsies was reported as the final pathological diagnosis of the case. A cytopathology quality control procedure was set, according to which cytology/pathology slides of each case were analyzed independently by two experienced cytologists/pathologists blinded to any other results or analysis. After they reported the results, 10% of the cytology/pathology slides that were reported consistently normal and all the slides that were reported to have cervical lesions by one or both of the cytologists/pathologists in the primary analysis will be reviewed by a third cytopathologist. The final diagnosis would be that agreed upon by at least two of the three cytopathologists.

### Statistical analysis

SPSS 25.0 software was used for all analyses. The consistency of BMRT and Cobas4800 for detecting hrHPV (referring to cases qualitatively positive of any HPV types), HPV-16 (referring to cases positive of HPV-16 singly or multiple types including HPV-16), HPV-18 (referring to cases positive of HPV-18 singly or multiple types including HPV-18), and the other 12 types of hrHPV in the pool (12-HPV pool) (referring to cases positive of another type (s) singly or multiply) was tested by Kappa coefficients. Student's *t*-test was used to compare the two assays for the mean CtV-Cobas and VL (lg)-BMRT of the 14 hrHPV, HPV-16, HPV-18, and 12-HPV pool. Pearson's or Spearman's correlation coefficient (r) was applied to analyze CtV-Cobas and VL (lg)-BMRT relative to the histology diagnosis, and linear regression lines were fitted in the scatterplot. All statistical tests were two-sided, and *P* < 0.05 was considered statistically significant.

## Results

### Characteristics of the study population

The average age of the 4,485 women included in this study is 45.4±7.2 years, with no significant difference from that of all the participants of CHIMUST. After excluding 189 cases that were positive for Cobas4800 and/or BMRT who did not return for colposcopy, two tests failed for Cobas4800, one test failed for cytology, and three had missing Cobas4800 data from the 1,495 HPV positives. A total of 1,300 positive cases were included in the analysis, with 2,990 negative cases, totaling 4,290 cases with complete data. Pathology diagnostics were performed on 1,258 cases, including 987 normal, 149 CIN 1, 74 CIN 2, 43 CIN 3, and 5 cervical cancers.

### Comparisons of type-specific hrHPV viral loads in the Cobas4800/BMRT HPV test

Among the 4,290 cases, 807 were positive for Cobas4800 HPV testing, with a rate of 18.8% (807/4290), and 745 were positive for BMRT HPV testing, with a rate of 17.4% (745/4,290). Of the 807 Cobas4800 positives, 185 were positive for HPV-16 and/or−18, and 682 were positive for the 12-HPV pool. Among the 745 BMRT positives, 140 were positive for HPV-16 and/or−18, and 645 were positive for the 12-HPV pool. The consistency of genotypes from Cobas4800 and BMRT for hrHPV overall, HPV-16, HPV-18, and 12-HPV pool was 94.9% (4,070/4,290, Kappa = 0.827), 99.1% (4,251/4,290, Kappa = 0.842), 99.6% (4,273/4,290, Kappa = 0.777), and 95.3% (4,089/4,290, Kappa = 0.821), respectively, indicating good consistency of the two assays.

Further analysis of all the cases with inconsistent testing results between the two assays (Table 1) showed that, when compared with the cases positive on Cobas4800 and BMRT, respectively, the mean CtVs of the cases negative by BMRT but positive on Cobas4800 for HPV-16 (*n* = 35), HPV-18 (*n* = 16), and 12-HPV pool (*n* = 119) are significantly higher, respectively. The mean VL (lg) of the cases negative for Cobas4800 but positive for BMRT for HPV-16 (*n* = 4), HPV-18 (*n* = 1), and 12-HPV pool (*n* = 82) is significantly lower, respectively. This indicates that the inconsistencies between the two assays are likely associated with the samples having comparatively lower viral loads.

**Table 1:** Genotype-specific CtV and VL (lg) of the HPV-positive samples.

**Result**		**HPV**−**16**			**HPV**−**18**			**12–HPV pool**		
		**Cobas+/**	**Cobas+/**	**Cobas–/**	**Cobas+/**	**Cobas+/**	**Cobas–/**	**Cobas+/**	**Cobas+/**	**Cobas–/**
		**BMRT+**	**BMRT–**	**BMRT+**	**BMRT+**	**BMRT–**	**BMRT+**	**BMRT+**	**BMRT–**	**BMRT+**
		**(*n* = 108)**	**(*n* = 35)**	**(*n* = 4)**	**(*n* = 30)**	**(*n* = 16)**	**(*n* = 1)**	**(*n* = 563)**	**(*n* = 119)**	**(*n* = 82)**
CtV–Cobas	range	20.5–40.3	25.8–40.4	–	23.0–39.5	28.2–39.8	–	17.6–40.0	25.9–40.0	–
	mean	28.53	35.87^a^	–	30.47	35.63^a^	–	30.20	35.82^a^	–
	95% CI	27.8–29.2	34.7–37.0	–	28.8–32.1	33.6–37.7	–	29.8–30.6	35.2–36.5	–
VL(lg)–BMRT	range	1.38–5.67	–	0.81	1.32–5.28	–	1.51	1.00–17.99	–	1.00–7.42
	mean	3.69	–	1.81	3.62	–		4.78	–	2.96^b^
	95% CI	3.48–3.89	–	2.48 2.67	3.23–4.01	–		4.58–4.97	–	2.72–3.19

a Compared with the CtV of the corresponding genotype Cobas (+) /BMRT (+), P < 0.001.

b Compared with the VL (lg) of the corresponding genotype Cobas (+) /BMRT (+), P < 0.001.

**Figure 2:**
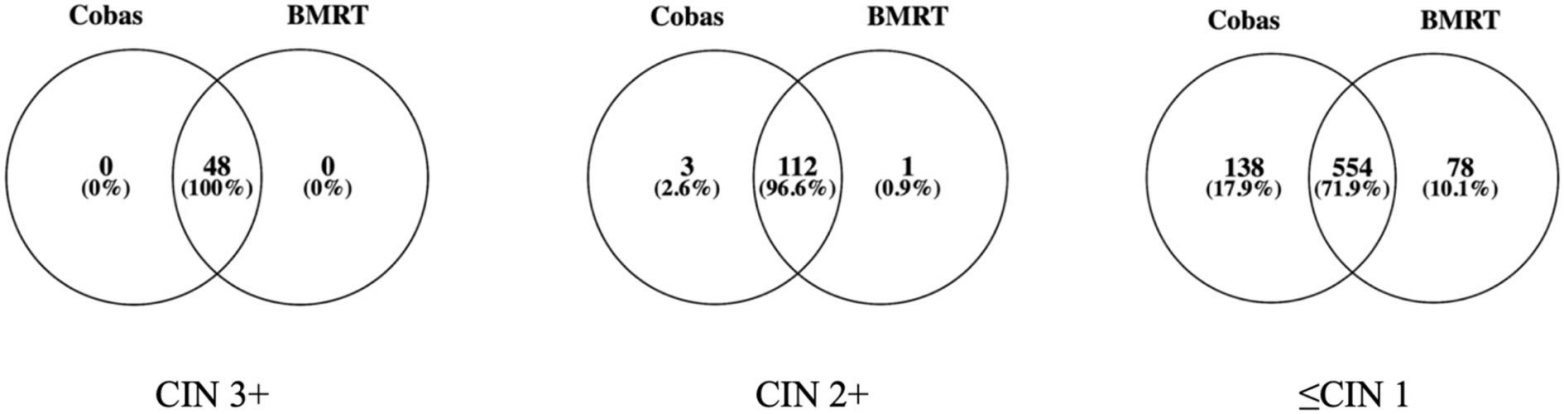
Per lesion-grade detection of Cobas4800 and BMRT (*n*; %).

Cobas4800 and BMRT detected 100% of the 48 CIN3+. Of the 122 CIN2+ cases, Cobas4800 and BMRT detected 115 (94.3% and 115/122) and 113 (92.6% and 113/122) cases, respectively, while among the 1,136 ≤CIN 1 cases, Cobas4800 detected 692 (60.9%, 692/1,136) and BMRT detected 632 (55.6% and 632/1136) (Figure 2).

### Correlation between CtV from Cobas4800 and VL (lg) per unit cells from BMRT

**Figure 3:**
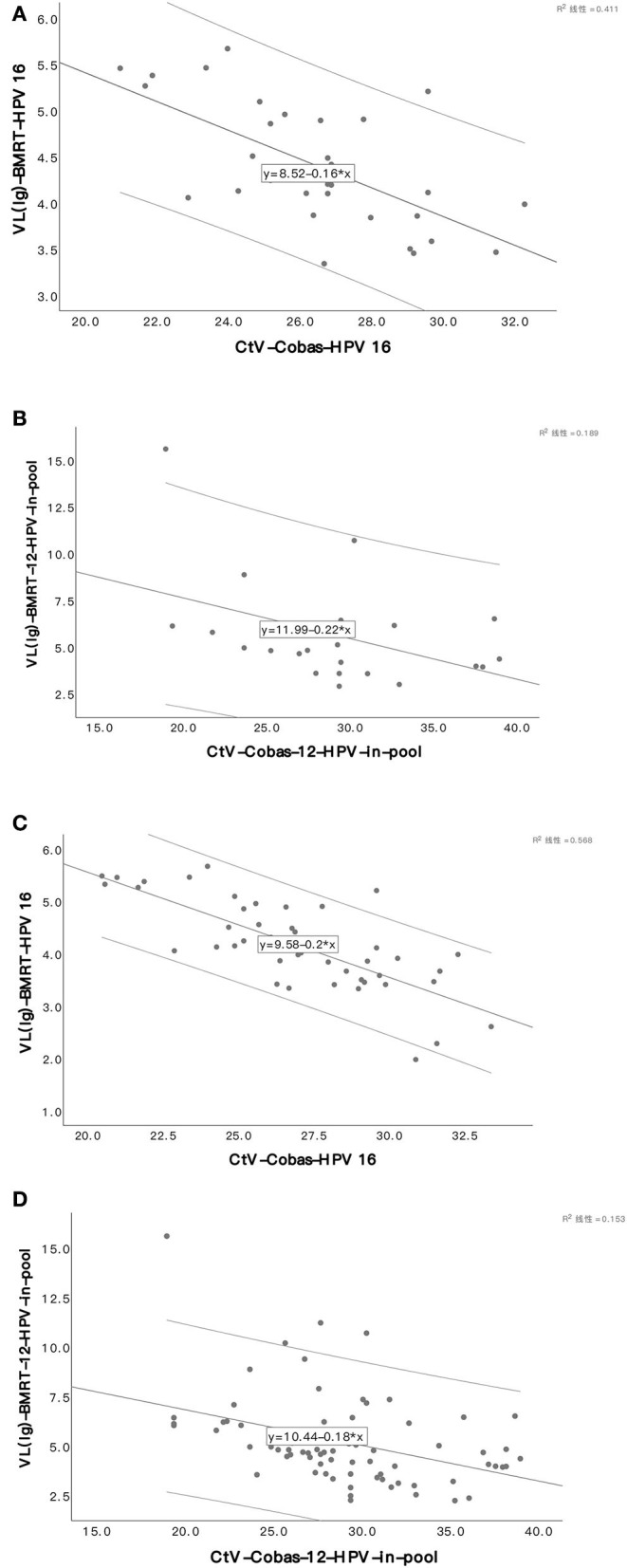
Scatter plot of viral load in two assays in CIN3+/CIN2+. HPV16 viral load in CIN3+ **(A)**. 12-HPV pool viral loads in CIN3+ **(B)**. HPV16 viral load in CIN2+ **(C)**. 12-HPV pool viral loads in CIN2+ **(D)**.

In an analysis of the HPV genotyping of Cobas4800 and BMRT, the two assays reported 32 HPV-16 positive and 23 12-HPV pool positive cases among the 48 CIN3+ cases, and 50 HPV-16 positives and 73 12-HPV pool positive cases among the 122 CIN2+ cases. The CtV from Cobas4800 and the VL (lg) from BMRT are highly correlated and follow linear regression. (Pearson's or Spearman's correlation coefficient (r): In CIN3+, r _HPV16_ = −0.641, *P* < 0.001; r _12−HPVpool_ = −0.343, *P* = 0.109; In CIN2+, r _HPV16_ = −0.754, *P* < 0.001; r _12−HPVpool_ = −0.429, *P* < 0.001). Correlation analyses were not conducted for CIN2+ cases positive for HPV-18 because we have only 1 CIN3+ and 3 CIN2+ cases positive for HPV-18 (Table 2, Figure 3).

**Table 2:** The number of positive cases in CIN2+/CIN3+.

**Pathological grade**	**HPV**−**16 (**+**)**		**HPV**−**18 (**+**)**		**12–HPV pool (**+**)**	
	**Cobas**	**BMRT**	**Cobas**	**BMRT**	**Cobas**	**BMRT**
CIN 2+	51(50)	51(50)	6(3)	4(3)	79(73)	74(73)
CIN 3+	32(32)	33(32)	1(1)	2(1)	25(23)	23(23)

(n), the number of cases positive in two assays.

## Discussion

Our study shows that the Cobas4800 and BMRT assays consistently detect the viral load of hrHPV overall, HPV-16, and 12-HPV pool from the physician-collected samples. A possible reason for the good consistency might be that the two assays are both based on real-time fluorescent PCR amplification technology, which needs to be demonstrated further. There were only a few cases with inconsistent results between the two assays, of which 3 (2.1%, 3/141) in the 141 Cobas (+) /BMRT (-) cases and 1 in the 79 (1.3%, 1/79) Cobas (-) /BMRT (+) cases were diagnosed as CIN2. All those CIN2 cases were reported positive in the 12-HPV pool with low viral loads. This fact indicates that most CIN2+ cases and all the CIN3+ cases are related to a medium or high viral load level in the specimens. We need further follow-up to demonstrate whether CIN2 with low viral load may become potentially regressive. Supportive to those indications are the known facts that HPV DNA integrating into the human genome is a vital event in cervical carcinogenesis (19); HPV-16 integration with comparatively higher viral load is more likely related to HSIL+; HPV-18 integration into the host genome was an independent risk factor (20). It is no doubt that HPV viral load from a physician-collected cervical sample for Cobas4800 HPV testing is preferable for the prediction of CIN2+ as that from self-collected vaginal samples (21).

Inconsistent measurement of viral loads in previous studies has been a barrier to its clinical application for a referral. Many investigators believe that it is difficult to make the per-sample number of cells similar. Since the number of cells in a sample could influence the circulation time of DNA amplification to give a quantification of viral load on a semi-quantitative HPV testing (22), homogenization of samples using standardized sampling is the basis for clinical application of CtV, which was what we did in CHIMUST.

With the samples collected by physicians using a standardized sampling technology, our team demonstrated that CtV from Cobas4800 was diversely correlated to the grading of cervical lesions caused by HPV-16 and 12-HPV pools and published relevant papers (12). In this analysis, we verified that diverse correlation by providing direct per unit cell viral loads (lg) from BMRT, which automatically calculates the number of cells in the sample based on the single copy of detectable genes and generates a normalized viral load; the viral copies of 21 HPV subtypes in 10,000 cells and provides an objective reference for clinical application. A comparison shows that, in CIN2+/CIN3+ cases positive for the two assays, CtVs from Cobas4800 for HPV-16 and 12-HPV pools are inversely correlated to the relevant viral loads (lg) per 10,000 cells from BMRT following a linear regression. The explanation for the comparatively lower correlation between CtV and viral loads (lg) for the 12-HPV pool may lie in the fact that we sum up the viral loads (lg) from BMRT from 12 hrHPV genotypes for comparison because Cobas4800 detects 12 hrHPV genotypes (31, 33, 35, 39, 45, 51, 52, 56, 58, 59, 66, and 68) in the pool. However, it is impossible to know if the cases positive for the 12-HPV pool in 2 assays are positive for the same genotype.

Many investigators paid attention to viral load in the prediction of CIN3 risks. In 1999, Swan et al. (23) first described the association between HPV viral load and cervical lesions. Hesselink et al. (24) believed that the viral load of HPV-16/-18/-31/-33 could not predict the risk of CIN2+/CIN3+ in HPV-positive patients. However, more studies have shown that hrHPV viral load can be used as an important indicator for persistent HPV infection, cervical lesions, and prognosis (25–28). Oyervides-Muñoz MA et al. (25) demonstrated a significant difference in HPV viral load between samples with persistent and transient HPV infections. Volpini LPB et al. (26) demonstrated that the viral load of the hrHPV among the types included in the 9-valent HPV vaccine was higher in the cases with cervical lesions. Luo et al. (27) reported that Hybrid Capture 2 (HC2) based on relative light units showed indirectly that viral load was positively associated with cervical lesions by grade. Consistent with Luo, Basu, P. et al. (28) further proposed that the viral load detected by HC2 could guide colposcopy because, in their study, 48.3% of CIN2+ and 80.4% of CIN3+ missed by colposcopy demonstrated intermediate or high viral loads. Compared with HPV-negative women, patients with high viral loads had a 46-fold increase in CIN2+, even when colposcopy appeared normal. Those facts strongly suggest that multiple punch biopsies should be performed on patients with an intermediate or high viral load to avoid missing high-grade squamous intraepithelial lesions (HSIL). Some scholars suggest that the cross-sectional viral load plays a limited role in predicting outcomes, but the change of HPV titers from at least two consecutive tests is more important (29, 30). The viral load of HPV-16 declining >2 logs across two tests indicates the virus may be cleared automatically (30). It appears that, based on the amount of scientific evidence, HPV viral load can be an important index for risk stratification to triage HPV-positive patients and a guide for the colposcopy exam.

Outstanding work in making viral load-related indicators as positive triage indexes includes Duan's study using HPV16/18 and ≤31 Ct value for 12-HPV pool to triage patients who were positive for HPV for Cobas4800 from 10,399 clinician-samples, who found that the sensitivity of this triage in the detection of CIN2+/CIN3+ was comparable with a triage based on HPV-16/-18 genotypes and cytology ≥ ASCUS for 12-HPV pool (12); Song's study using genotypes of HPV-16/-18 and ≤33.7 CtV of 12-HPV pool to triage patients who were positive for hrHPV for Cobas4800 from 10,498 self-sampling samples, who demonstrated higher sensitivity and specificity of this triage for CIN2+/CIN3+ than using only genotypes for HPV-16/-18 (8); and Zhang's study to decide Cobas-CtVs of hrHPV, 12-HPV pool, HPV-16, and HPV-16/18 as the cutoffs to separate cases with CIN3+ risk <4% and ≥4%, who reported that Cobas-CtV 33.2 and 29.6 could be the cutoffs for hrHPV and 12-HPV pool, respectively. Our results show that CtVs from Cobas4800 are inversely consistent with the viral loads in per-unit of cells from BMRT and provide further explanation for Zhang's accomplishments. This finding provides direct evidence of the role of virus load in positive triage as a molecular index.

Our work is meaningful because it reduces barriers to primary screening and positive triage for cervical cancer prevention by using molecular evidence from a single sample and eliminating the requirement for follow-up visits. The HPV test is effective for cervical cancer screening, but its comparatively lower specificity makes secondary screening for triage of the HPV positives required by most guidance. Although HPV genotypes have been adopted by many guidelines to be the triage index for HPV-16/-18, cytology is also recommended as the secondary screening for women who are positive for the 12-HPV pool (7). Secondary screening with cytology would not only rely on high resources for qualification but also require multiple visits for diagnosis, which would increase the economic burden of screening women and the follow-up losses. When referable, molecular tests make the triage more objective, reproducible, higher throughput, less follow-up loss, and more applicable in lower-resource areas.

The limitation of this study is that we have to sum the viral loads of the 12-HPV pool together for analysis because Cobas4800 cannot distinguish the other 12 types of hrHPV in a pool, but BMRT can. We have no evidence to confirm whether this summation creates a bias in comparing the CtV with BMRT viral load for the 12-HPV pool.

In conclusion, when the samples were obtained by standardized sampling, the CtV from Cobas4800 and the viral loads (lg) per unit cell from the BMRT were well correlated. CtV-Cobas merits additional clinical promotion and validation.

## Data availability statement

The raw data supporting the conclusions of this article will be made available by the authors, without undue reservation.

## Ethics statement

The studies involving human participants were reviewed and approved by the Ethics Committee from Peking University Shenzhen Hospital and Cleveland Clinic Institutional Review Board. The patients/participants provided their written informed consent to participate in this study.

## Author contributions

RW, LW, and JB conceived and designed the study. QY, HD, and XQ analyzed, interpreted the patient data and drafted the manuscript. WD, LG, CL, CW, CG, and YZ contributed to the data collection and quality control. All authors contributed to the article and approved the submitted version.

## Funding

The study was supported by the Shenzhen High-level Hospital Construction Fund (YBH2019-260), the Shenzhen Key Medical Discipline Construction Fund (Grant No. SZXK027), and the Sanming Project of Medicine in Shenzhen (Grant No. SZSM202011016).

## Conflict of interest

Author JB was employed by Preventive Oncology International, Inc. The remaining authors declare that the research was conducted in the absence of any commercial or financial relationships that could be construed as a potential conflict of interest.

## Publisher's note

All claims expressed in this article are solely those of the authors and do not necessarily represent those of their affiliated organizations, or those of the publisher, the editors and the reviewers. Any product that may be evaluated in this article, or claim that may be made by its manufacturer, is not guaranteed or endorsed by the publisher.
